# Circulating branched-chain amino acids and long-term risk of obesity-related cancers in women

**DOI:** 10.1038/s41598-020-73499-x

**Published:** 2020-10-06

**Authors:** Deirdre K. Tobias, Aditi Hazra, Patrick R. Lawler, Paulette D. Chandler, Daniel I. Chasman, Julie E. Buring, I-Min Lee, Susan Cheng, JoAnn E. Manson, Samia Mora

**Affiliations:** 1 Division of Preventive Medicine, Department of Medicine, Brigham and Women’s Hospital and Harvard Medical School, 900 Commonwealth Avenue, Boston, MA 02215 USA; 2 Department of Nutrition, Harvard T.H. Chan School of Public Health, Boston, MA USA; 3 Peter Munk Cardiac Centre, University Health Network, and Heart and Stroke Richard Lewar Centre of Excellence in Cardiovascular Research, University of Toronto, Toronto, ON Canada; 4 Department of Epidemiology, Harvard T.H. Chan School of Public Health, Boston, MA USA; 5 Division of Cardiovascular Medicine, Department of Medicine, Center for Lipid Metabolomics, Brigham and Women’s Hospital and Harvard Medical School, Boston, MA USA; 6 Barbra Streisand Women’s Heart Center and Smidt Heart Institute at Cedars-Sinai, Los Angeles, CA USA; 7 Mary Horrigan Connors Center for Women’s Health and Gender Biology, Brigham and Women’s Hospital and Harvard Medical School, Boston, MA USA

**Keywords:** Cancer epidemiology, Predictive markers, Cancer, Endocrine system and metabolic diseases

## Abstract

Obesity is a risk factor for > 13 cancer sites, although it is unknown whether there is a common mechanism across sites. Evidence suggests a role for impaired branched-chain amino acid (BCAAs; isoleucine, leucine, valine) metabolism in obesity, insulin resistance, and immunity; thus, we hypothesized circulating BCAAs may be associated with incident obesity-related cancers. We analyzed participants in the prospective Women’s Health Study without a history of cancer at baseline blood collection (N = 26,711, mean age = 54.6 years [SD = 7.1]). BCAAs were quantified via NMR spectroscopy, log-transformed, and standardized. We used Cox proportional regression models adjusted for age, race, smoking, diet, alcohol, physical activity, menopausal hormone use, Body Mass Index (BMI), diabetes, and other risk factors. The endpoint was a composite of obesity-related cancers, defined per the International Agency for Research on Cancer 2016 report, over a median 24 years follow-up. Baseline BMI ≥ 30 kg/m^2^ compared with BMI 18.5–25.0 kg/m^2^ was associated with 23% greater risk of obesity-related cancers (n = 2751 events; multivariable HR 1.23, 95% CI 1.11–1.37). However, BCAAs were not associated with obesity-related cancers (multivariable HR per SD = 1.01 [0.97–1.05]). Results for individual BCAA metabolites suggested a modest association for leucine with obesity-related cancers (1.04 [1.00–1.08]), and no association for isoleucine or valine (0.99 [0.95–1.03] and 1.00 [0.96–1.04], respectively). Exploratory analyses of BCAAs with individual sites included positive associations between leucine and postmenopausal breast cancer, and isoleucine with pancreatic cancer. Total circulating BCAAs were unrelated to obesity-related cancer incidence although an association was observed for leucine with incident obesity-related cancer.

## Introduction

Excess body weight is a recognized risk factor for at least 13 cancer sites, contributing up to 9% of the cancer burden in developed countries^1^. Overweight and obesity-related cancers disproportionately affect women, accounting for 55% of cancers diagnosed in US women and 24% of those in men. Furthermore, while the incidence of malignancies overall has declined over the past 2 decades, obesity-related cancers continue to rise^2^. As the prevalence of overweight and obesity in the US and globally increases, a downstream surge in obesity-related cancers is inevitable without effective strategies for prevention. The metabolic consequences of excess adiposity are diverse and diffuse, yet its role in mediating cancer risk is poorly understood. Thus, approaches that seek to identify the relevance of obesity-related pathways to cancer incidence and survival are warranted to understand the mechanisms underlying the global obesity-cancer link. In addition, interventions and therapies targeting upstream modifiable obesity-related mechanisms may provide powerful broad-spectrum strategies for the prevention of several cancer sites among high-risk individuals^3^.

Recent applications of high-throughput plasma metabolite profiling technologies (i.e., metabolomics) in observational human studies illustrate individual metabolites and metabolomic networks associated with excess body weight and obesity. Among them, circulating branched-chain amino acids (BCAAs; isoleucine, leucine, and valine) have emerged as strongly and positively associated with adiposity^4–7^, cardiometabolic traits^8^, and type 2 diabetes^9^. A cross-sectional study identified BCAAs were up to 20% higher among participants with obesity vs. lean; further, a BCAA metabolomic score indicative of dysregulated BCAA catabolism was strongly correlated with insulin resistance (r = 0.58, p < 0.0001)^6^. In parallel, a growing body of research has also identified circulating BCAA metabolite concentrations as positively associated with some site-specific cancers, including pancreas^10^ and postmenopausal breast, both of which are obesity-related cancer sites^11^. In vitro evidence also supports a role for BCAAs in breast cancer development in human breast epithelial cells, with research demonstrating that a knock-in of a single allele *BRCA1* mutation led to increased BCAAs^12^. Leucine, in particular, has been implicated in pancreatic tumor growth in an obese mouse model^13^, and poor breast cancer survival^14^.

It is plausible that obesity-related impairments in BCAA catabolism, reflected through elevated BCAA metabolites in circulation, are upstream drivers of multiple obesity-related cancer sites. However, no prior research has prospectively investigated all BCAAs collectively and comprehensively in relation to the spectrum of obesity-related cancer sites. Better understanding of obesity-related mechanisms agnostic to cancer tissue type has the potential to influence several downstream malignancies representing a significant portion of the obesity-related cancer burden^3^. Thus, we hypothesized that baseline plasma total and individuals BCAAs would be positively associated with a composite endpoint of obesity-related cancers in the longitudinal Women’s Health Study (WHS) cohort of 39,876 US women with over two decades of prospective follow-up. Secondarily, we evaluated the relationship between BCAAs with the common site-specific obesity-related cancers including digestive tract cancers, postmenopausal breast, uterus, ovary, renal cell, and multiple myeloma.

## Methods

### Study population

We conducted this study in the WHS, an ongoing longitudinal cohort study of 39,876 female US health professionals aged ≥ 45 years without history of prior cancer (except non-melanoma skin cancer) or cardiovascular disease at enrollment. The WHS began as a randomized placebo-controlled trial of low-dose aspirin, β-carotene, and vitamin E for the primary prevention of cardiovascular disease and cancer and ran from 1993 to 2004 (NCT00000479), after which it was converted to an observational cohort with ongoing follow-up^15,16^. Overall there was no effect of the randomized interventions on cancer incidence^17–19^. The WHS continues to follow participants annually on an observational basis. There were 28,345 women who participated in the voluntary baseline blood sample collection, of which 20,392 were fasting (< 8 h since last eating at blood draw). Blood samples were collected and shipped on ice via overnight courier to the central laboratory where they were processed and stored at − 170 °C in vapor liquid nitrogen. The WHS questionnaires captured demographics, health status, reproductive history, and lifestyle characteristics.

For this analysis we included women participating in the blood sample collection, excluding those with missing information on baseline height and weight from which body mass index (BMI kg/m^2^) was derived, with baseline BMI < 18.5 kg/m^2^, or with a diagnosis of cardiovascular disease or cancer prior to baseline. Written informed consent was obtained from all participants and the study protocol was approved by the Institutional Review Board of the Brigham and Women’s Hospital (Boston, MA) and all research was performed in accordance with relevant guidelines and regulations.

### BCAA laboratory methods

WHS baseline blood samples were assayed to quantify a targeted panel of metabolites, which included isoleucine, leucine, and valine. Briefly, aliquots of the EDTA plasma samples were shipped on dry ice blinded to outcome status to LipoScience, Inc, now LabCorp (Raleigh, NC). Isoleucine, leucine, and valine were measured by proton nuclear magnetic resonance (^1^H NMR) spectroscopy using a 400 MHz NMR platform, as described for the NMR LipoProfile IV test^20,21^. The NMR spectra were deconvoluted using proprietary software with models containing reference spectra for BCAAs and the derived BCAA signal amplitudes were converted to µmol/L. The intra- and inter-assay coefficients of variation for the assays were: isoleucine 5.9–6.1%, leucine 4.5–4.9%, and valine 1.5–2.1%.

### Ascertainment of incident cancer cases

Questionnaires were sent to participants to ascertain any newly diagnosed endpoints on an annual basis. Medical records were obtained with participants’ written consent and reviewed by an endpoints committee. Medical record review was completed for 95% of self-reported cancer cases and confirmation among those with available records was 82%^17^. In the present analysis we include confirmed invasive cancer cases through December 31, 2018. We classified obesity-related cancers based on the 2016 IARC report of 13 cancer sites or types with sufficient strength of evidence for an association with body fatness, including: adenocarcinoma of the esophagus, gastric cardia, colon and rectum, liver, gallbladder, pancreas, postmenopausal breast, corpus uteri, ovary, renal cell, meningioma, and multiple myeloma^1^. Thyroid cancers were not included given the potential for considerable surveillance bias in their detection. Deaths were identified from surveillance of family members, postal authorities, or National Death Index, with nearly 100% mortality follow-up^22^. Cancer-specific deaths were confirmed against medical records, the National Death Index, death certificates by a physician committee.

### Assessment of covariates

Participants reported several characteristics related to health, lifestyle, reproductive history, medical diagnoses, family history of cancer, current medication use, and other factors via questionnaires. Usual diet was assessed using a 131-item semi-quantitative food frequency questionnaire (FFQ)^23,24^, from which we derived the Alternative Health Eating Index 2010 (aHEI-2010) dietary quality score for each individual, as previously described^25^. Physical activity was captured on the baseline questionnaire as the average time per week spent engaged in recreational activity domains (e.g., walking, running, bicycling), flights of stairs climbed daily, and usual walking pace^26^. Participants self-reported a physician diagnosis of type 2 diabetes, high blood pressure or high cholesterol. Hypertension was defined as self-report of a physician diagnosis, past or current antihypertensive treatment, systolic blood pressure ≥ 140 mmHg, or diastolic blood pressure ≥ 90 mmHg^27^, and high cholesterol was defined as self-report of a physician diagnosis or use of cholesterol-lowering medications.

### Statistical analysis

WHS participants with baseline blood samples with BCAA measures were eligible for our analysis (Supplemental Fig. S1). We calculated total BCAAs as the sum of the three individual metabolite levels. We transformed the individual and total BCAA metabolite concentrations to the natural log (ln) scale to improve normal distribution and standardized to mean of 0 and standard deviation (SD) of 1. Metabolites were analyzed continuously per SD and in quintiles. Cancers with a date of diagnosis < 2 years from the baseline were excluded to minimize the potential influence from underlying malignancy at blood draw. We used ANOVA to compare the baseline characteristics of participants across quintiles of total plasma BCAA concentration. We conducted a multivariable Cox proportional hazards regression model to confirm the association between baseline BMI with the composite of incident obesity-related cancers. We conducted sensitivity analyses excluding non-fasting samples (< 8 h since last eating at blood draw).

Cox proportional hazards regression models were used with follow-up from the date of WHS randomization to date of first invasive cancer diagnosis, death, or December 31, 2018, whichever came first. Models were adjusted for age (years) and original WHS randomization (aspirin, vitamin E, beta carotene vs. placebo) (Model 1). In Model 2, we additionally adjusted for fasting status at blood draw (≥ 8 h since last eating), and cancer risk factors at baseline including postmenopausal status (yes, no), hormone therapy use (never, past, current, missing), Caucasian race/ethnicity, smoking status (never, former, current), AHEI diet quality score (continuous), alcohol intake (never/rarely, 1–3 drinks/month, 1–6 drinks/week, ≥ 1 drinks/day), total physical activity (MET-hours/week; continuous), and baseline histories of hypertension or high cholesterol. In Model 3 we further adjusted for BMI (kg/m^2^; continuous). Missing indicator variables were included for missing covariate data (< 1% of data for all variables except fasting status was missing for 5.1% samples), where applicable. In sensitivity analyses we excluded participants with baseline type 2 diabetes, excluded cancers diagnosed within the first 5 years from baseline blood draw, excluded non-fasting samples (< 8 h since last eating), or further adjusted for type 2 diabetes and cardiometabolic biomarkers previously assayed from the baseline blood samples, including lipoprotein insulin resistance (LPIR) score, triglycerides, high-sensitivity C-reactive protein (hsCRP), and HbA1c, to evaluate whether BCAAs were associated with cancers independent of these other plausible obesity-related cancer pathways.

We also evaluated secondary outcomes of interest including deaths from obesity-related cancers and site-specific cancers for digestive tract cancers (colorectal, pancreas, other combined), postmenopausal breast, uterus, ovary, renal cell, and multiple myeloma. Fatal obesity-related cancers were classified if cause of death was confirmed to be any cancer after an incident cancer diagnosis of an obesity-related cancer. We also performed analyses stratified by baseline BMI above/below 25 kg/m^2^ and tested for a statistical interaction with likelihood ratio tests comparing the multivariable models with and without inclusion of the multiplicative interaction term. The Cox proportional hazard assumption was tested through the inclusion of a cross product term for BCAA and time (years from baseline blood draw); this assumption was met, with no indication for a violation. We used SAS Version 9.3 software (SAS Institute, Cary, NC) for all analyses.

## Results

Of the 28,345 women participating in the baseline blood collection there were 26,711 eligible for inclusion in this analysis (Supplemental Fig. S1), and 19,249 (72%) were fasting. Individual plasma BCAAs were moderately correlated, ranging from r = 0.51 for isoleucine with leucine and r = 0.67 for leucine with valine (Supplemental Table S2). Baseline characteristics are given in Table 1 by quintiles of baseline plasma total BCAAs. Total BCAAs were correlated with BMI (r = 0.31) and the prevalence of obesity (BMI ≥ 30 kg/m^2^) was consistently higher across BCAA quintiles with Q1 = 7.8%, Q2 = 10.3%, Q3 = 14.8%, Q4 = 21.8%, and Q5 = 33.0%. Lower intake of alcohol, lower physical activity, and modestly lower AHEI diet quality score were also correlated with higher circulating BCAAs. Obesity-related comorbidities were prevalent among higher BCAA levels, including type 2 diabetes, high cholesterol, and high blood pressure.

**Table 1:** Baseline characteristics of WHS participants by quintiles of plasma total branched-chain amino acids (BCAAs).

	Total plasma branched-chain amino acid (BCAA) quintiles				
	Q1	Q2	Q3	Q4	Q5
Participants, N	5342	5342	5342	5342	5343

	Values are means (standard deviations) or %, unless otherwise specified				
Age, years	54.3 (7.1)	54.7 (7.2)	54.8 (7.0)	54.8 (7.0)	54.5 (6.8)
**Body Mass Index, kg/m**^**2**^	24.1 (3.8)	24.9 (4.1)	25.7 (4.6)	26.8 (5.0)	28.4 (5.7)
Normal weight (18.5–25.0)	69.2	61.6	53.1	43.0	30.8
Overweight (25.0–29.9)	23.0	28.1	32.1	35.3	36.2
Obese (≥ 30.0)	7.8	10.3	14.8	21.8	33.0
Postmenopausal	51.0	54.2	55.9	55.8	52.7
Current postmenopausal hormone use	42.7	43.8	44.4	42.7	39.7
Caucasian race/ethnicity	95.3	95.4	95.0	94.1	93.0
**Smoking status**					
Never	48.8	52.0	52.1	53.2	52.7
Past	38.5	37.1	37.0	34.7	35.8
Current, < 15 cig/day	4.6	4.4	4.2	3.7	4.3
Current, ≥ 15 cig/day	8.1	6.3	6.5	7.1	6.9
**Alcohol**					
Rarely/never	38.6	40.4	42.8	45.5	52.0
1–3 drinks/month	12.5	14.4	13.3	13.2	13.4
1–6 drinks/week	35.4	33.3	33.7	32.3	27.6
≥ 1 drink/day	13.5	11.9	10.3	9.0	7.0
AHEI diet quality score (range 10–100)	49.0 (9.7)	48.7 (9.5)	48.5 (9.4)	48.1 (9.3)	47.6 (9.4)
Total physical activity, MET-hrs/wk	16.6 (20.2)	15.7 (18.5)	14.9 (17.8)	12.5 (16.6)	12.5 (16.6)
History of type 2 diabetes	0.6	0.9	1.2	2.1	7.9
History of high cholesterol	23.5	27.1	29.6	32.1	34.0
History of hypertension	18.3	21.3	22.8	27.7	34.2
**Randomized treatment assignment**					
Aspirin	49.7	50.2	49.8	49.4	51.2
Beta carotene	49.7	50.5	49.8	49.8	49.4
Vitamin E	49.9	49.6	49.9	51.1	49.7

Plasma metabolite levels, umol/L	Median (interquartile range)				
Total BCAA	309 (284, 325)	361 (350, 372)	401 (392, 412)	447 (434, 461)	528 (499, 577)
Isoleucine	35 (27, 43)	43 (36, 51)	50 (42, 58)	58 (49, 67)	76 (64, 90)
Leucine	96 (83, 108)	118 (107, 128)	132 (121, 143)	147 (136, 159)	177 (161, 195)
Valine	173 (159, 185)	200 (189, 211)	220 (209, 231)	242 (230, 255)	284 (265, 308)

*AHEI* Alternative healthy eating index score, *MET* metabolic equivalent of task.

We observed 4308 total incident primary cancers, of which 2751 were classified as obesity-related sites, over 23.6 years median follow-up. Compared with women with a normal BMI (18.5–24.9 kg/m^2^), having obesity (BMI ≥ 30.0 kg/m^2^) at baseline was associated with a 23% greater risk of developing an incident obesity-related cancer in the multivariable-adjusted model (HR 1.23, 95% CI 1.11, 1.37); but not with the remaining cancers not classified as obesity-related (HR 0.90, CI 0.78, 1.05).

For the primary composite outcome of incident obesity-related cancers, continuous BCAAs were modestly associated with a higher cancer risk adjusting for age and randomized treatment assignment (Table 2; per SD HR 1.04, CI 1.00, 1.08). This association was null after multivariable-adjustment for cancer risk factors including BMI (per SD HR 1.01, CI 0.97, 1.05). In the minimally-adjusted model leucine (per SD HR 1.06, CI 1.02, 1.10) was associated with a higher risk of incident obesity cancers while isoleucine and valine were not. After multivariable-adjustment leucine remained positively associated with cancer risk (HR 1.04, CI 1.00, 1.08). Comparing higher BCAAs across quintiles with the lowest reference quintile suggested a dose response only for leucine (p for trend = 0.043), although the difference between top and bottom quintiles was not significant with additional adjustment for BMI (HR 1.10, CI 0.97, 1.24). We did not observe associations for BCAAs with obesity-related cancer death (Table 3).

**Table 2:** Multivariable-adjusted Cox proportional hazards models (95% CIs) for quintiles of baseline plasma BCAAs and incidence of obesity-related cancers in the WHS.

	Continuous (per SD)	Quintiles of Plasma BCAA Metabolites					*p-*trend
		Q1	Q2	Q3	Q4	Q5	
	HR (95% CI)	[reference]	HR (95% CI)	HR (95% CI)	HR (95% CI)	HR (95% CI)	
**Total BCAAs**							
N cases	2751	545	515	509	609	573	
Model 1	1.04 (1.00, 1.08)	1.00	0.93 (0.82, 1.05)	0.91 (0.81, 1.03)	1.10 (0.98, 1.24)	1.07 (0.96, 1.21)	0.029
Model 2	1.04 (1.00, 1.09)	1.00	0.93 (0.82, 1.05)	0.91 (0.80, 1.02)	1.10 (0.98, 1.23)	1.08 (0.96, 1.22)	0.030
Model 3	1.01 (0.97, 1.05)	1.00	0.92 (0.81, 1.03)	0.88 (0.78, 0.99)	1.04 (0.93, 1.18)	1.00 (0.88, 1.13)	0.47
**Isoleucine**							
N cases	2751	572	517	516	575	571	
Model 1	1.01 (0.97, 1.05)	1.00	0.90 (0.80, 1.01)	0.90 (0.80, 1.02)	1.01 (0.90, 1.13)	1.04 (0.92, 1.16)	0.28
Model 2	1.01 (0.97, 1.05)	1.00	0.89 (0.79, 1.01)	0.90 (0.80, 1.02)	1.01 (0.90, 1.13)	1.04 (0.92, 1.17)	0.31
Model 3	0.99 (0.95, 1.03)	1.00	0.89 (0.79, 1.00)	0.88 (0.78, 0.99)	0.97 (0.86, 1.09)	0.97 (0.86, 1.09)	0.89
**Leucine**							
N cases	2751	528	503	547	581	592	
Model 1	1.06 (1.02, 1.10)	1.00	0.95 (0.84, 1.07)	1.03 (0.91, 1.16)	1.11 (0.98, 1.24)	1.15 (1.02, 1.30)	0.0024
Model 2	1.06 (1.02, 1.10)	1.00	0.95 (0.84, 1.07)	1.02 (0.90, 1.15)	1.10 (0.98, 1.24)	1.15 (1.02, 1.30)	0.003
Model 3	1.04 (1.00, 1.08)	1.00	0.94 (0.83, 1.06)	1.00 (0.89, 1.13)	1.07 (0.95, 1.20)	1.10 (0.97, 1.24)	0.043
**Valine**							
N cases	2751	539	524	559	542	587	
Model 1	1.03 (0.99, 1.07)	1.00	0.96 (0.85, 1.08)	1.01 (0.90, 1.14)	0.99 (0.88, 1.11)	1.10 (0.98, 1.24)	0.10
Model 2	1.03 (0.99, 1.07)	1.00	0.96 (0.85, 1.08)	1.01 (0.90, 1.14)	0.99 (0.88, 1.11)	1.10 (0.98, 1.24)	0.096
Model 3	1.00 (0.96, 1.04)	1.00	0.95 (0.84, 1.07)	0.98 (0.87, 1.10)	0.93 (0.83, 1.06)	1.01 (0.89, 1.14)	0.95

*Model 1* is adjusted for age and randomized treatment assignment (aspirin, vitamin E, beta carotene); *Model 2* additionally adjusts for risk factors at baseline blood draw including fasting status (≥ 8 h since last eating), postmenopausal status (yes, no), hormone therapy use (never, past, current, missing), Caucasian race/ethnicity (yes, no), smoking status (never, former, current), AHEI diet quality score (continuous), alcohol intake (never/rarely to 1–3 drinks/month, 1–6 drinks/week, ≥ 1 drinks/day), total physical activity (MET-hours/week; continuous), history of high cholesterol, and history of high blood pressure; *Model 3* additionally adjusts for BMI (kg/m^2^; continuous).

*SD* standard deviation.

BCAAs SD = 1.0; isoleucine SD = 1.0; leucine SD = 1.0; valine SD = 1.0.

**Table 3:** Multivariable-adjusted Cox proportional hazards models (95% CIs) for quintiles of baseline plasma BCAAs and incidence of obesity-related cancer deaths (n = 476 deaths) in the WHS.

	Continuous (per SD)	Quintiles of plasma BCAA metabolites					*p*-trend
		Q1	Q2	Q3	Q4	Q5	
	HR (95% CI)	[reference]	HR (95% CI)	HR (95% CI)	HR (95% CI)	HR (95% CI)	
**Multivariable-adjusted model**^a^							
Total BCAAs	1.03 (0.93, 1.14)	1.00	0.97 (0.73, 1.29)	0.89 (0.66, 1.19)	1.06 (0.80. 1.41)	1.07 (0.79, 1.43)	0.55
Isoleucine	1.02 (0.93, 1.12)	1.00	0.94 (0.71, 1.24)	1.01 (0.76, 1.33)	0.86 (0.64, 1.15)	1.06 (0.80, 1.41)	0.93
Leucine	1.03 (0.93, 1.15)	1.00	0.98 (0.73, 1.31)	1.00 (0.75, 1.34)	0.99 (0.74, 1.33)	1.23 (0.93, 1.63)	0.21
Valine	1.01 (0.92, 1.11)	1.00	1.12 (0.84, 1.49)	1.09 (0.81, 1.45)	1.05 (0.78, 1.41)	1.07 (0.79, 1.46)	0.80

^a^Cox model is adjusted for age and randomized treatment assignment (aspirin, vitamin E, beta carotene), fasting status (≥ 8 h since last eating), postmenopausal status (yes, no), hormone therapy use (never, past, current, missing), Caucasian race/ethnicity (yes, no), smoking status (never, former, current), AHEI diet quality score (continuous), alcohol intake (never/rarely to 1–3 drinks/month, 1–6 drinks/week, ≥ 1 drinks/day), total physical activity (MET-hours/week; continuous), history of high cholesterol, history of high blood pressure, and BMI (kg/m^2^; continuous).

**Figure 1:**
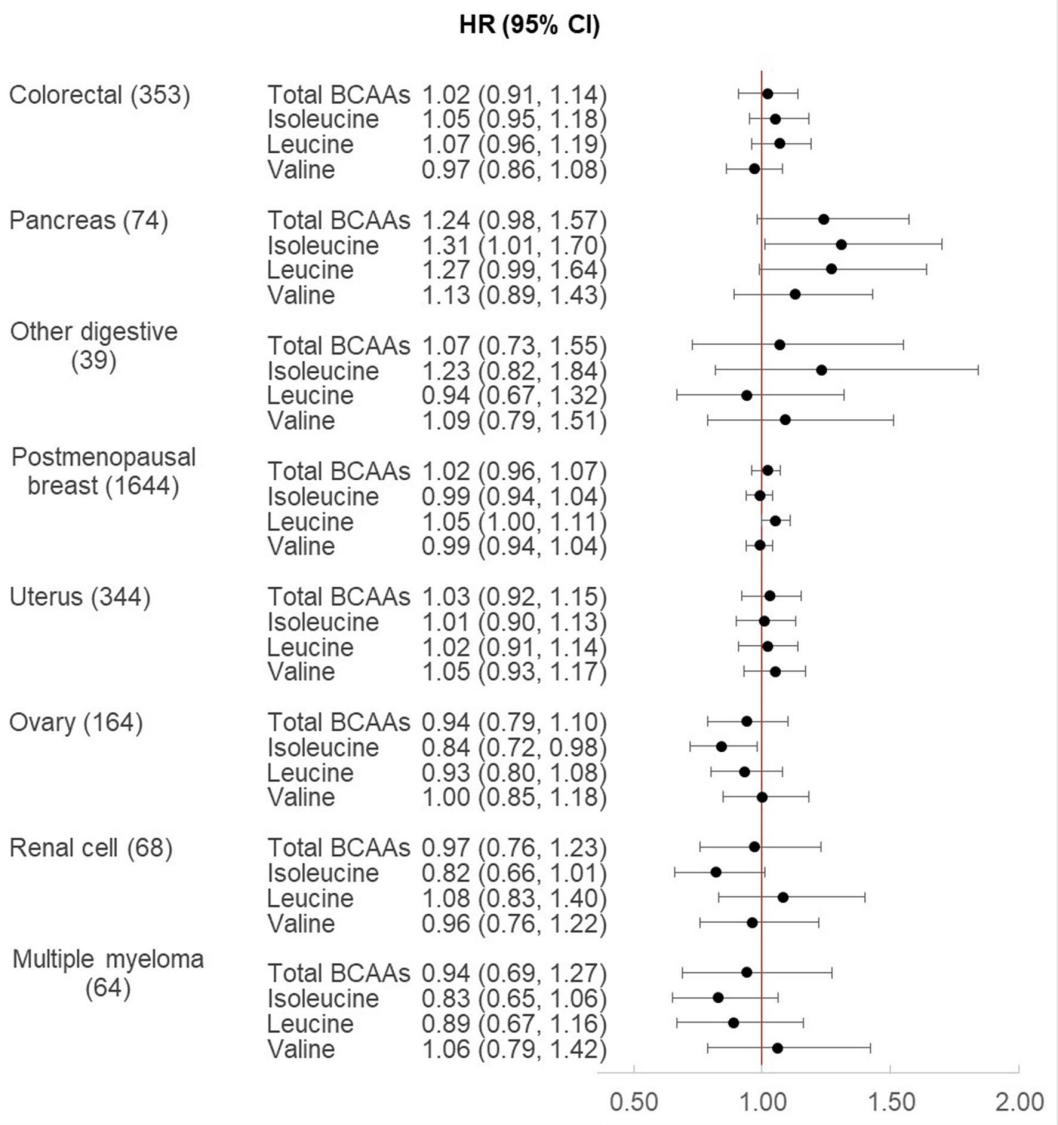
Plasma branched chain amino acid metabolites and risk of incident obesity-related cancers in women. The Cox model is adjusted for age and randomized treatment assignment (aspirin, vitamin E, beta carotene), fasting status (≥ 8 h since last eating), postmenopausal status (yes, no), hormone therapy use (never, past, current, missing), Caucasian race/ethnicity (yes, no), smoking status (never, former, current), AHEI diet quality score (continuous), alcohol intake (never/rarely to 1–3 drinks/month, 1–6 drinks/week, ≥ 1 drinks/day), total physical activity (MET-hours/week; continuous), history of high cholesterol, history of high blood pressure, and BMI (kg/m^2^; continuous).

Results varied for the exploratory analyses of continuous plasma BCAAs with individual cancer sites (Fig. 1) and according to baseline BMI (< 25 vs. ≥ 25 kg/m^2^) in Supplemental Table S2. Total BCAAs were not associated with any of the individual obesity-related cancer sites independent of BMI and multivariable adjustment for other cancer risk factors. BCAAs were positively associated with pancreas cancer (74 cases), with a significant association observed for isoleucine (multivariable-adjusted HR per SD 1.31, CI 1.01, 1.70), and borderline associations for total BCAAs (multivariable-adjusted HR per SD = 1.24, CI 0.98, 1.57) and leucine (multivariable-adjusted HR per SD = 1.27, CI 0.99, 1.64), while valine was not associated (multivariable-adjusted HR per SD = 1.13, CI 0.89, 1.43). These associations appeared stronger for the subgroup of women with BMI in the normal range at blood draw, although p-values for interaction were non-significant. BCAAs were not associated with colorectal cancers overall, except for isoleucine which was positively associated with colorectal cancers among women with overweight or obesity (multivariable-adjusted HR per SD = 1.18, CI 1.01, 1.37; p-interaction = 0.012). Only leucine was modestly associated with a higher postmenopausal breast cancer risk (1644 cases; multivariable-adjusted HR per SD = 1.05, CI 1.00, 1.11). Isoleucine, but not leucine or valine, was inversely associated with risks of ovary and renal cell cancers, particularly among women with normal BMI (p-interaction < 0.05 for both). BCAAs were not associated with uterus cancers or multiple myelomas.

When we restricted the analysis to fasting samples only (Supplemental Table S3), the finding for leucine were strengthened with total obesity-related cancers (multivariable-adjusted HR per SD = 1.08, CI 1.02, 1.13) and postmenopausal breast cancer (multivariable-adjusted HR per SD = 1.09, CI 1.02, 1.16), as were the associations for isoleucine and leucine with pancreas cancer (multivariable-adjusted HR per SD = 1.45, CI 1.03, 2.04; HR per SD = 1.40, CI 1.01, 1.95, respectively). Results were largely unchanged after excluding participants with prevalent diabetes at blood draw, and adjusting for other obesity-related cardiometabolic traits (Supplemental Table S3).

## Discussion

Despite their correlation with body weight, total levels of circulating BCAAs were not associated with the composite endpoint of obesity-related cancers. We hypothesized that BCAAs, as proximal markers of excess adiposity, may represent a shared underlying pathway for these cancer sites. In this cohort of US women with long-term follow-up we did not observe an association between total BCAAs with overall obesity-related cancer risk, although some heterogeneity was noted by individual BCAAs and by cancer site. In particular, leucine was associated with obesity-related cancers. In hypothesis-generating analyses, we also observed site-specific associations that may reflect differences in BCAA-related pathways for individual cancer sites including pancreas, colorectal, postmenopausal breast, ovary, and renal cell.

A previous prospective study identified leucine among the top metabolomic predictors for postmenopausal breast cancer over a median follow-up of 6.7 years from baseline blood draw in the Prostate, Lung, Colorectal, and Ovarian Cancer Screening cohort (621 cases), which is consistent with our finding; however we did not replicate their positive associations for isoleucine and valine^11^. We also observed baseline plasma isoleucine was associated with a 30% higher risk of incident cancers of the pancreas (HR per SD = 1.31, 95% CI 1.01, 1.70) that was twofold higher among those with normal BMI at baseline (HR per SD = 1.61, CI 1.10, 2.36). Previously, a pooled nested case–control analysis (454 cases) of men and women identified leucine and isoleucine as the strongest metabolite predictors of incident pancreatic adenocarcinoma (leucine: OR per SD = 1.28, 95% CI 1.11, 1.48; isoleucine: OR 1.28, 95% CI 1.13, 1.46) diagnosed a mean 8.7 years after blood draw^10^. Strongest findings were observed for cancers within 5 years of diagnosis. Similarly, our association between BCAAs with pancreatic cancers was attenuated and no longer significant when cancers diagnosed within 5 years from baseline blood draw were excluded, consistent with the hypothesis that BCAA levels may increase in pancreatic cancers as a result of cancer-related tissue protein breakdown^10^. This finding was replicated in a prospective cohort study in a Japanese population (170 cases), which reported leucine per SD OR 1.31 (95% CI 1.05, 1.63) with pancreatic cancer^28^. Prospective studies identifying pre-diagnosis metabolites associated with site-specific cancers are otherwise sparse, and further research and replication are warranted.

Accumulating evidence implicates excess body weight as a risk factor for at least 13 cancer sites^1^; however, evidence for common mechanisms across these sites is sparse. Plausible drivers include chronic inflammation, insulin resistance, dyslipidemia, and endocrine alterations, which have the potential to impact tumor incidence and progression irrespective of tissue site^29,30^. BCAAs are strongly correlated with body weight-related and long-term risk of incident cardiometabolic disease including type 2 diabetes, making these a compelling target of investigation for obesity-related cancers^8,31^. BCAAs are essential amino acids, derived from a variety of dietary protein sources; however, their concentrations in circulation during fasting typically correlate poorly with their dietary intake^32,33^. Circulating BCAA concentrations more likely reflect rate of BCAA oxidation, insulin resistance, or in some instances, skeletal muscle catabolism (wasting)^34^. In the present analysis we observed noticeably different associations by metabolite and cancer site, consistent with some prior site-specific mechanistic research. In vitro evidence supports BCAAs as a factor for breast cancer development in human breast epithelial cells, demonstrating that a knock-in of a single allele BRCA1 mutation increased BCAA levels^12^. Leucine supplementation promotes protein biosynthesis in humans by activating complex 1 of the mammalian target of rapamycin (mTORC1), which is a critical regulator of T cell proliferation, differentiation, and function^35,36^. Of the three BCAAs, leucine has strongest effect on mTOR activation and protein synthesis in several tissues, including skeletal muscle, which may explain its association with total obesity-related cancers and cancer mortality in our present analysis, while isoleucine and valine were not associated^37^. Knockdown experiments demonstrated that LLGL2 scaffolding protein regulates amino-acid induced activation of the mTOR pathway in estrogen receptor positive (ER+) breast cancer cells, and high expression of these leucine transporters were correlated with poor survival in ER+ breast cancer patients treated with tamoxifen^14^. Leucine supplementation increased phosphorylation of mTOR in lean mice, while it increased circulating glucose in mice with obesity, with these different mechanisms leading to increases in pancreatic tumor growth for both groups^13^. These data suggest that leucine may promote some site-specific cancers through a variety of mechanisms, although evidence for many cancer sites is sparse. Further, evidence for protective mechanisms unique to isoleucine upstream of ovary and renal cell cancers is limited and replication of these unexpected findings is needed.

Strengths of this analysis include the large prospective study with cohort-wide ascertainment of metabolites, and the prospective adjudication of incident cancer cases allowing us to evaluate BCAAs in relation to many cancer sites simultaneously in a pan-cancer approach. Long-term follow-up allowed us to maintain power after exclusion of cases occurring early in follow-up to minimize the influence of undiagnosed cancers on the baseline metabolite levels (i.e., reverse causation). The use of a state-of-the-art NMR metabolomic platform benefits from reduced measurement error. We adjusted for validated lifestyle measures and obesity-related comorbidities collected in the WHS baseline questionnaires to minimize potential confounding by cancer risk factors.

We also acknowledge limitations in this analysis. Repeated measures would minimize the within-person variability over time; however, blood samples were only drawn at baseline from WHS participants and thus random within-person error leading to an underestimate of the true associations is possible. Second, BMI may misclassify women with differing magnitudes of abdominal adiposity, if regional fat distribution is a driver of the obesity-cancer relationship. The analyses for the site-specific obesity-related cancers are likely to be underpowered after correction for multiple comparisons, thus we are cautious in drawing conclusions for any apparent between-site heterogeneity, and replication for these findings are needed. Finally, BCAAs may be correlated with other relevant obesity-related metabolites or pathways that we do not account for here. However, adjusting for biomarkers of dyslipidemia, glucose metabolism, and inflammation in sensitivity analyses did not change results.

## Conclusions

Identifying common biological pathways across obesity-related cancer sites may isolate upstream novel targets for prevention of several cancer sites simultaneously. However, total BCAAs are unlikely to represent one such pathway underlying the relationship between excess adiposity across obesity-related cancer sites. Metabolite-specific associations, such as the positive associations for leucine and isoleucine with specific cancer sites are supported by previous prospective studies and mechanistic research. The positive association for BCAAs with cancers of the pancreas was also previously observed and may reflect preexisting pathology at blood draw leading to elevations in circulating BCAAs.

## Supplementary information

Supplementary Information.

## Data Availability

The datasets generated during and/or analyzed during the current study are available to researchers on reasonable request from the WHS data usage review committee.
