# Supplementary material for: Circulating branched-chain amino acids and long-term risk of obesity-related cancers in women
Source: Sci Rep. 2020 Oct 6;10:16534. doi: 10.1038/s41598-020-73499-x (PMC7539150; doi:10.1038/s41598-020-73499-x)
Supplement: Supplementary file 1 — Supplementary Information. [file 41598_2020_73499_MOESM1_ESM.docx]

**Circulating branched-chain amino acids and long-term risk of obesity-related cancers in women**

Running title: BCAAs and obesity-related cancers

Deirdre K. Tobias, ScD (1, 2), Aditi Hazra (1), Patrick R. Lawler, MD (3), Paulette D. Chandler, MD MPH (1), Daniel I. Chasman, PhD (1), Julie E. Buring, ScD (1, 4), I-Min Lee, ScD (1, 4), Susan Cheng, MD (5, 6), JoAnn E. Manson, MD DrPH (1, 4, 7), Samia Mora, MD, MHS (1, 5)

**Supplemental Table 1.** Pearson correlation coefficients for baseline metabolites* and body mass index (BMI, kg/m^2^).

| **Total study population** | Total BCAAs | Isoleucine | Leucine | Valine | BMI |
| --- | --- | --- | --- | --- | --- |
| Total BCAAs | 1.00 | 0.74 | 0.86 | 0.93 | 0.31 |
| Isoleucine |  | 1.00 | 0.51 | 0.60 | 0.24 |
| Leucine |  |  | 1.00 | 0.67 | 0.20 |
| Valine |  |  |  | 1.00 | 0.32 |
| BMI |  |  |  |  | 1.00 |

*Metabolites are log-transformed and standardized.

**Supplemental Table 2.** Plasma branched chain amino acid metabolites and risk of incident obesity-related cancers, obesity-related cancer mortality, and site-specific cancers in the WHS, stratified by baseline BMI.

|  | **Baseline BMI** | |
| --- | --- | --- |
|  | **Normal**  **(BMI < 25.0 kg/m^2^)**  **N=13,762** | **Overweight/Obese**  **(BMI ≥ 25.0 kg/m^2^)**  **N=12,949** |
|  | **Multivariable-Adjusted HR (95% CI)^** | **Multivariable-Adjusted HR (95% CI)^** |
| **Total obesity-related cancers** |  |  |
| N cases | 1,325 | 1,426 |
| Total BCAAs | 1.02 (0.96, 1.08) | 1.01 (0.95, 1.06) |
| Isoleucine | 0.96 (0.90, 1.01) | 1.01 (0.96, 1.07) |
| Leucine | 1.06 (1.00, 1.12) | 1.02 (0.97, 1.08) |
| Valine | 1.01 (0.95, 1.07) | 0.99 (0.93, 1.04) |
|  |  |  |
| **Colorectal** |  |  |
| N cases | 159 | 194 |
| Total BCAAs | 0.97 (0.82, 1.15) | 1.06 (0.91, 1.22) |
| Isoleucine | 0.94 (0.81, 1.09) | 1.18 (1.01, 1.37) |
| Leucine | 1.07 (0.91, 1.26) | 1.06 (0.92, 1.22) |
| Valine | 0.93 (0.79, 1.10) | 0.99 (0.85, 1.15) |
|  |  |  |
| **Pancreas** |  |  |
| N cases | 37 | 37 |
| Total BCAAs | 1.44 (1.01, 2.05) | 1.04 (0.77, 1.40) |
| Isoleucine | 1.61 (1.10, 2.36) | 1.05 (0.77, 1.44) |
| Leucine | 1.45 (0.99, 2.11) | 1.09 (0.79, 1.51) |
| Valine | 1.26 (0.88, 1.82) | 1.00 (0.74, 1.34) |
|  |  |  |
| **Other digestive** |  |  |
| N cases | 11 | 28 |
| Total BCAAs | 1.16 (0.84, 1.61) | 1.01 (0.61, 1.67) |
| Isoleucine | 1.03 (0.63, 1.67) | 1.31 (0.77, 2.23) |
| Leucine | 1.30 (0.88, 1.93) | 0.83 (0.57, 1.23) |
| Valine | 1.10 (0.81, 1.50) | 1.07 (0.69, 1.64) |
|  |  |  |
| **Postmenopausal breast** |  |  |
| N cases | 832 | 812 |
| Total BCAAs | 1.03 (0.96, 1.11) | 0.99 (0.92, 1.06) |
| Isoleucine | 1.00 (0.93, 1.07) | 0.97 (0.90, 1.05) |
| Leucine | 1.07 (1.00, 1.15) | 1.03 (0.96, 1.10) |
| Valine | 1.01 (0.93, 1.08) | 0.97 (0.90, 1.04) |
|  |  |  |
| **Uterus** |  |  |
| N cases | 125 | 219 |
| Total BCAAs | 1.01 (0.83, 1.23) | 1.05 (0.92, 1.20) |
| Isoleucine | 0.98 (0.82, 1.16) | 1.03 (0.90, 1.19) |
| Leucine | 0.99 (0.83, 1.19) | 1.04 (0.90, 1.19) |
| Valine | 1.06 (0.87, 1.29) | 1.05 (0.91, 1.21) |
|  |  |  |
| **Ovary** |  |  |
| N cases | 97 | 67 |
| Total BCAAs | 0.84 (0.66, 1.08) | 1.07 (0.88, 1.30) |
| Isoleucine | 0.71 (0.59, 0.85) | 1.15 (0.94, 1.42) |
| Leucine | 0.87 (0.71, 1.06) | 1.03 (0.85, 1.25) |
| Valine | 0.95 (0.75, 1.20) | 1.06 (0.85, 1.32) |
|  |  |  |
| **Renal cell** |  |  |
| N cases | 26 | 42 |
| Total BCAAs | 0.86 (0.55, 1.35) | 1.01 (0.76, 1.36) |
| Isoleucine | 0.68 (0.48, 0.97) | 0.92 (0.72, 1.18) |
| Leucine | 1.08 (0.70, 1.67) | 1.06 (0.76, 1.48) |
| Valine | 0.85 (0.55, 1.30) | 1.02 (0.76, 1.37) |
|  |  |  |
| **Multiple myeloma** |  |  |
| N cases | 37 | 27 |
| Total BCAAs | 1.16 (0.77, 1.76) | 0.72 (0.48, 1.08) |
| Isoleucine | 0.86 (0.60, 1.25) | 0.78 (0.58, 1.05) |
| Leucine | 1.08 (0.72, 1.62) | 0.72 (0.52, 0.98) |
| Valine | 1.29 (0.85, 1.97) | 0.84 (0.60, 1.18) |

^Cox model is adjusted for age and randomized treatment assignment (aspirin, vitamin E, beta carotene), fasting status (≥8 hours since last eating), postmenopausal status (yes, no), hormone therapy use (never, past, current, missing), Caucasian race/ethnicity (yes, no), smoking status (never, former, current), AHEI diet quality score (continuous), alcohol intake (never/rarely to 1-3 drinks/month, 1-6 drinks/week, ≥1 drinks/day), total physical activity (MET-hours/week; continuous), history of high cholesterol, history of high blood pressure, BMI (kg/m^2^; continuous).

**Supplemental Table 3.** Sensitivity analyses for plasma branched chain amino acid metabolites and risk of incident obesity-related cancers, obesity-related cancer mortality, and site-specific cancers in the WHS.

|  | **Exclude non-fasting samples (<8 hours since last eating)**  **N=19,249** | **Exclude prevalent T2D at baseline**  **N=23,412** | **Exclude cancers <5 years follow-up** | **Additional adjustment for cardiometabolic biomarkers** |
| --- | --- | --- | --- | --- |
|  | **Multivariable-adjusted HR^ (95% CI)** | **Multivariable-adjusted HR^ (95% CI)** | **Multivariable-adjusted HR^ (95% CI)** | **Multivariable-adjusted HR^ (95% CI)** |
| **Total obesity-related cancers** |  |  |  |  |
| N cases | 2,025 | 2,684 | 2,360 | 2,733 |
| Total BCAAs | 1.04 (0.99, 1.09) | 1.01 (0.97, 1.05) | 1.01 (0.97, 1.06) | 1.02 (0.97, 1.06) |
| Isoleucine | 1.00 (0.95, 1.05) | 0.98 (0.94, 1.02) | 0.99 (0.94, 1.03) | 0.99 (0.95, 1.03) |
| Leucine | 1.08 (1.02, 1.13) | 1.04 (1.00, 1.08) | 1.04 (0.99, 1.08) | 1.04 (1.00, 1.08) |
| Valine | 1.01 (0.96, 1.06) | 0.99 (0.95, 1.03) | 1.00 (0.95, 1.04) | 1.00 (0.96, 1.04) |
|  |  |  |  |  |
| **Colorectal** |  |  |  |  |
| N cases | 264 | 344 | 294 | 352 |
| Total BCAAs | 1.03 (0.89, 1.18) | 1.02 (0.91, 1.14) | 1.06 (0.94, 1.19) | 1.01 (0.90, 1.13) |
| Isoleucine | 1.08 (0.94, 1.23) | 1.06 (0.95, 1.18) | 1.07 (0.94, 1.20) | 1.04 (0.93, 1.16) |
| Leucine | 1.08 (0.95, 1.23) | 1.07 (0.96, 1.19) | 1.08 (0.96, 1.22) | 1.07 (0.96, 1.19) |
| Valine | 0.95 (0.83, 1.10) | 0.96 (0.85, 1.07) | 1.02 (0.90, 1.15) | 0.94 (0.84, 1.07) |
|  |  |  |  |  |
| **Pancreas** |  |  |  |  |
| N cases | 55 | 73 | 62 | 74 |
| Total BCAAs | 1.31 (0.96, 1.78) | 1.27 (1.00, 1.62) | 1.15 (0.87, 1.51) | 1.14 (0.90, 1.46) |
| Isoleucine | 1.45 (1.03, 2.04) | 1.33 (1.02, 1.74) | 1.11 (0.91, 1.65) | 1.20 (0.93, 1.55) |
| Leucine | 1.40 (1.01, 1.95) | 1.33 (1.02, 1.72) | 1.29 (0.96, 1.73) | 1.23 (0.96, 1.57) |
| Valine | 1.11 (0.83, 1.48) | 1.15 (0.90, 1.46) | 0.98 (0.75, 1.28) | 1.02 (0.80, 1.31) |
|  |  |  |  |  |
| **Other digestive** |  |  |  |  |
| N cases | 31 | 37 | 39 | 39 |
| Total BCAAs | 0.96 (0.59, 1.56) | 1.03 (0.69, 1.53) | 1.07 (0.73, 1.55) | 1.07 (0.73, 1.58) |
| Isoleucine | 1.14 (0.71, 1.85) | 1.14 (0.77, 1.67) | 1.23 (0.82, 1.84) | 1.24 (0.86, 1.87) |
| Leucine | 0.89 (0.62, 1.30) | 0.93 (0.66, 1.31) | 0.94 (0.67, 1.32) | 0.95 (0.68, 1.34) |
| Valine | 0.97 (0.64, 1.47) | 1.08 (0.76, 1.52) | 1.09 (0.79, 1.51) | 1.10 (0.78, 1.55) |
|  |  |  |  |  |
| **Postmenopausal breast** |  |  |  |  |
| N cases | 1211 | 1608 | 1418 | 1,634 |
| Total BCAAs | 1.05 (0.98, 1.12) | 1.01 (0.96, 1.06) | 1.02 (0.96, 1.08) | 1.02 (0.97, 1.08) |
| Isoleucine | 1.01 (0.94, 1.07) | 0.98 (0.93, 1.04) | 1.00 (0.94, 1.05) | 1.00 (0.95, 1.06) |
| Leucine | 1.09 (1.02, 1.16) | 1.05 (1.00, 1.10) | 1.05 (1.00, 1.11) | 1.05 (1.00, 1.11) |
| Valine | 1.01 (0.94, 1.07) | 0.98 (0.93, 1.04) | 0.99 (0.94, 1.05) | 1.00 (0.95, 1.05) |
|  |  |  |  |  |
| **Uterus** |  |  |  |  |
| N cases | 252 | 335 | 302 | 342 |
| Total BCAAs | 1.03 (0.89, 1.19) | 1.02 (0.91, 1.15) | 1.02 (0.90, 1.15) | 1.06 (0.94, 1.19) |
| Isoleucine | 1.02 (0.89, 1.17) | 1.01 (0.90, 1.12) | 0.99 (0.88, 1.11) | 1.04 (0.92, 1.16) |
| Leucine | 1.02 (0.89, 1.17) | 1.02 (0.91, 1.13) | 1.03 (0.91, 1.16) | 1.03 (0.92, 1.15) |
| Valine | 1.03 (0.89, 1.19) | 1.04 (0.92, 1.16) | 1.03 (0.91, 1.17) | 1.08 (0.96, 1.22) |
|  |  |  |  |  |
| **Ovary** |  |  |  |  |
| N cases | 116 | 163 | 131 | 161 |
| Total BCAAs | 1.01 (0.83, 1.21) | 0.94 (0.79, 1.11) | 0.94 (0.78, 1.13) | 0.94 (0.79, 1.13) |
| Isoleucine | 0.81 (0.67, 0.99) | 0.83 (0.71, 0.97) | 0.84 (0.70, 1.00) | 0.85 (0.72, 1.00) |
| Leucine | 1.00 (0.83, 1.20) | 0.93 (0.80, 1.08) | 0.94 (0.79, 1.13) | 0.94 (0.81, 1.09) |
| Valine | 1.07 (0.89, 1.29) | 1.01 (0.85, 1.19) | 0.99 (0.82, 1.19) | 1.00 (0.84, 1.20) |
|  |  |  |  |  |
| **Renal cell** |  |  |  |  |
| N cases | 48 | 61 | 56 | 66 |
| Total BCAAs | 1.00 (0.73, 1.38) | 0.94 (0.73, 1.22) | 0.85 (0.67, 1.08) | 0.81 (0.62, 1.05) |
| Isoleucine | 0.75 (0.58, 0.97) | 0.78 (0.63, 0.96) | 0.81 (0.65, 1.00) | 0.73 (0.60, 0.89) |
| Leucine | 1.14 (0.79, 1.64) | 1.05 (0.80, 1.39) | 0.90 (0.70, 1.16) | 0.94 (0.73, 1.21) |
| Valine | 1.02 (0.76, 1.37) | 0.96 (0.75, 1.23) | 0.88 (0.69, 1.12) | 0.82 (0.62, 1.07) |
|  |  |  |  |  |
| **Multiple myeloma** |  |  |  |  |
| N cases | 47 | 62 | 57 | 64 |
| Total BCAAs | 1.09 (0.74, 1.58) | 0.92 (0.68, 1.26) | 0.91 (0.66, 1.25) | 0.97 (0.71, 1.33) |
| Isoleucine | 0.99 (0.73, 1.35) | 0.81 (0.64, 1.03) | 0.80 (0.62, 1.03) | 0.86 (0.66, 1.10) |
| Leucine | 0.99 (0.70, 1.40) | 0.88 (0.66, 1.15) | 0.88 (0.67, 1.16) | 0.88 (0.66, 1.17) |
| Valine | 1.18 (0.82, 1.70) | 1.06 (0.78, 1.43) | 1.02 (0.75, 1.40) | 1.12 (0.83, 1.52) |

^Cox model is adjusted for age and randomized treatment assignment (aspirin, vitamin E, beta carotene), fasting status (≥8 hours since last eating), postmenopausal status (yes, no), hormone therapy use (never, past, current, missing), Caucasian race/ethnicity (yes, no), smoking status (never, former, current), AHEI diet quality score (continuous), alcohol intake (never/rarely to 1-3 drinks/month, 1-6 drinks/week, ≥1 drinks/day), total physical activity (MET-hours/week; continuous), history of high cholesterol, history of high blood pressure, BMI (kg/m^2^; continuous)

**Supplemental Figure 1.** Analysis inclusion/exclusion criteria in WHS

WHS Randomized

N=39,876

WHS Blood Cohort

N=28,345

WHS Analytical Cohort

N=26,711

Exclusions:

No BCAA measures – n=911

BMI missing – n=345

BMI <18.5 kg/m^2^ – n=617

History of CVD – n=4

History of cancer – n=36

Cancers <2 year f/up – n=450
